# Editorial: Global excellence in brain-computer interfaces: Europe

**DOI:** 10.3389/fnhum.2023.1333272

**Published:** 2023-11-29

**Authors:** Andrej M. Savić, Pietro Aricò

**Affiliations:** ^1^School of Electrical Engineering, University of Belgrade, Belgrade, Serbia; ^2^Department of Computer, Control, and Management Engineering Antonio Ruberti, Sapienza University of Rome, Rome, Italy

**Keywords:** BCI, stroke, feature extraction, gaming, ergonomics, biomarkers, neurofeedback

Brain-computer interfaces (BCIs) measure brain activity and translate it into commands for a computer or machine, enabling users to control devices using only their thoughts. In February 2023, Nature Electronics Journal announced that BCIs were selected as the technology of the year (Drew, 2023). It has been fifty years since the term “brain-computer interface” was introduced, but the path to clinical and commercial success is still uncertain. Nevertheless, BCIs are a rapidly evolving technology with the potential to revolutionize human-machine interaction, a pursuit embraced by an increasing number of startup companies utilizing various technologies, including: sensors, signal processing methods, machine learning algorithms, and devices, or software applications designed for control (Soekadar et al., 2023).

BCI applications and use case scenarios are diverse but still constrained to several application domains: replacing functions that were lost due to injury or disease [e.g., communication in severely disabled (Savić et al., 2021; Vansteensel et al., 2023)]; restoring the lost functions [e.g., though controlled electrical stimulation of muscles in paralysis (Benabid et al., 2019)]; improving affected functions [e.g., BCI-based therapeutic interventions post-stroke (Nojima et al., 2022)]; enhancing the functions [e.g., neuromodulation of attention, memory, workload, motor skills (Sciaraffa et al., 2022; Novičić and Savić, 2023)]; and using BCI technology as research tools to study brain functions (Brunner et al., 2015). BCIs may not only serve the medical field and healthcare and could be applied in other areas, such as entertainment, gaming, education, ergonomics, marketing, and more (Falk et al., 2023; Soekadar et al., 2023).

This Research Topic comprises papers addressing trending topics relevant to the BCI field, such as the state of the art and future directions of BCIs for rehabilitation, feature extraction methods for BCI applications, home-based BCI intervention for pain treatment, theoretical models of decision-making in BCI applications, systems applicable in hybrid BCIs for neuroergonomics, electrophysiological biomarkers for early diagnosis of neurological diseases, and the exploration of cognitive mechanisms involved in human-computer interaction. All of these topics are closely related to innovative approaches and applications of BCI technologies.

Vidaurre et al. conducted an extensive literature review of various challenges related to the design and development of neural interfaces for BCI-based stroke rehabilitation. The authors analyzed the current bibliographic evidence of the effects of neurofeedback in functional motor rehabilitation after a stroke. They described the important aspects that should be considered when applying BCIs to restore motor control, such as dealing with noisy signals in realistic clinical environments, understanding voluntary and involuntary compensatory movement correlates, implementing feature extraction and classification approaches, adopting strategies for increasing usability and decreasing calibration time, utilizing different sensory feedback modalities for effective stimulation of afferent pathways including proprioceptive and tactile feedback, and immersive and multisensory feedback. They also discussed combinations with physiotherapy to generalize and exploit the rehabilitation effects of BCIs, considering the mental and physical states of the patients and emphasizing the importance of user involvement and acceptance. This study also reviewed the brain correlates of impairment and recovery, as well as cortico-muscular correlates in hybrid BCI control.

Hekmatmanesh et al. introduced a novel method for extracting discriminative patterns from electroencephalography (EEG) related to desired actions. They focused on an automatic identification method based on the largest Lyapunov exponent (LLE) chaotic feature to control a robotic bionic hand, with the classification task directed toward EEG motor imagery tasks, including hand opening and making a fist. In this study, the main constant input parameters of the LLE, named the false nearest neighbor, and mutual information, were parameterized and then optimized using the Water Drop (WD) and Chaotic Tug of War (CTW) optimizers. The experimental part of the study involved 21 healthy subjects performing motor imagery tasks. Offline methods were employed to assess the classification accuracy. The results showed that the CTW, with an average accuracy of 72.3%, outperformed both the traditional LLE and the optimized LLE using the WD optimizer. The study concluded that the traditional LLE required enhancement through optimization methods and that the CTW approximation method has the potential for more efficient solutions compared to the WD method. The overall significance of this work lies in the introduction of novel features based on complexity measures of motor imagery-related EEG into the field of EEG-based movement classification.

Birch et al. introduced a home-based EEG neurofeedback intervention for the management of chronic pain. Neurofeedback systems, utilizing the same methods and neurotechnology as BCIs, can help some patients manage chronic pain by normalizing maladaptive brain activity via online neuromodulation of certain EEG features. The objective of their study was to assess the efficacy and safety of a novel home-based neurofeedback device for managing chronic pain by modifying specific EEG activity. They conducted a prospective, single-arm, proof-of-concept study involving 16 adults with chronic pain. A neurofeedback device was provided to each participant, and 32–48 training sessions were completed over 8-week periods. The primary outcome measure was self-reported pain while the assessment of central sensitization, sleep quality, affective symptoms, change in quality of life, adverse events during use and EEG correlations with symptoms were secondary outcomes. Results showed that 11 subjects reported pain relief following neurofeedback training, while 8 reporting clinically significant improvements. Central sensitization symptoms improved by a third, sleep quality by almost 50%, anxiety reduced by 40%, and quality of life improved at final follow-up for 13 participants. The 69% of participants who upregulated relative alpha power reported improved pain, and those who downregulated beta power reported improved pain, reduced anxiety and depression scores. Authors conclude that home-based BCI training is well tolerated and may provide relief for sufferers of chronic pain and its associated symptoms.

Heskebeck et al. addressed the multi-armed bandit (MAB) problem and reviewed its implications in the field of BCI. MAB models an agent (decision-maker) seeking to optimize its actions to maximize expected rewards while having only partial knowledge of expected rewards for competing actions, gaining new knowledge after taking an action. Thus, the agent must navigate the exploration vs. exploitation trade-off problem within BCI systems, especially in the online setting, where properties of different choices may be only partially known but improve with more data. The MAB framework provides a structured approach for designing and analyzing BCI systems. The authors reviewed different aspects of interest for successfully understanding and potentially employing the MAB framework in BCIs, including algorithms for solving MAB problems, the current use of MAB in BCI, improving BCI calibration using MAB, and in real-time implementations of P300 speller. They also discussed the potential future use, such as addressing attention selection, providing data for transfer learning, and determining the optimal stopping time for the BCI calibration.

Valls-Serrano et al. explored the relationship between cognition and expertise in the context of video game technology, specifically examining whether visuospatial working memory and attention control differentiate experts, regular players, and non-players of the video game League of Legends. The study aimed to analyze cognitive differences in a sample of 80 participants across three expertise groups. The results confirmed that experts performed significantly better than regular players and non-video game players in the working memory test, and significant differences were found between players and non-video game players in the attention test. The study addressed four main domains of executive functions related to goal-directed behavior (spatial working memory, attention control, switching, and inhibition), with the overall findings indicating that experts exhibited superior spatial working memory and attention control, with no significant differences in switching and inhibition abilities. These results have important implications for human-computer interaction and BCI interventions based on serious games.

Balaji et al. proposed a study in the field of passive BCIs (Arico et al., 2017). In particular, they presented the development of a human digital twin (HDT) capable of simulating a control room operator's behavior, even during various abnormal situations, using the ACT-R (Adaptive Control of Thought-Rational) cognitive architecture. The system mimics a human operator monitoring the process and intervening during abnormal situations. The authors conducted 426 trials to assess the HDT's ability to handle disturbance rejection tasks. They validated the human digital twin by comparing it with the eye gaze behavior of 10 human subjects involved in 110 similar disturbance rejection tasks. The overall results revealed that the human digital twin's cognitive capabilities are comparable to those of human operators in the explored task. The authors discussed the possibilities of further robustness evaluation of their method, aligned with recent trends in neuroergonomics, using EEG data for workload estimation or tracking the learning progress of operators.

Perez-Valero et al. described an automated approach for the detection of Alzheimer's disease (AD) from resting-state EEG. This study addressed the crucial problem of early AD detection, aiming to control disease progression and delay intellectual decline. Many current detection techniques are costly, inaccessible, or invasive, relying on laborious manual analysis, which delays medical treatment. The authors conducted a preliminary evaluation of a fully-automated approach for AD detection based on an automated classification pipeline applied to resting-state EEG. The study involved 26 participants from three groups: mild AD, mild cognitive impairment (MCI-non-AD), and healthy controls. Automated data-driven algorithms were used to reject EEG artifacts, followed by spectral, complexity, and entropy feature extraction. Two binary classification problems were tested: mild AD vs. controls and MCI-non-AD vs. controls, through leave-one-subject-out cross-validation. The preliminary results were comparable to the best reported in the literature, suggesting that AD detection could be automatically achieved through automated processing and commercial EEG systems. This research aligns with recent trends in combining non-invasive electrophysiological recordings with state-of-the-art signal processing and machine learning techniques to discover novel biomarkers for neurological disorders (Petrusic et al., 2022; Mitrović et al., 2023).

To conclude, this collection aims to shed light on the recent progress made across the entire breadth of the BCI field and reflect on the future challenges faced by researchers across borders. All of the articles presented showed relevant ideas in various applications areas of BCIs. The editors are delighted to introduce this compilation of articles to the BCI field and its interconnected scientific communities. They anticipate that these contributions will support researchers in pushing forward the advancements of BCI and its diverse applications.

## Author contributions

AS: Conceptualization, Writing—original draft, Writing—review & editing. PA: Writing—review & editing.
